# Comparative Evaluation of Hydrofluoric Acid Etchants on a 3D-Printed Lithium Disilicate-Filled Polymer Resin

**DOI:** 10.3390/polym18212748

**Published:** 2026-11-04

**Authors:** Silvia Rojas-Rueda, Fabio Luiz Andretti, Carlos A. Jurado, Rene Garcia-Contreras, Jose Vivaldi, Abdullah Almansour, Hamad Algamaiah, Abdulrahman Alshabib

**Affiliations:** 1 School of Dental Medicine, Ponce Health Sciences University, Ponce 00716, Puerto Rico; 2 Division of Operative Dentistry, Department of General Dentistry, College of Dentistry, University of Tennessee Health Science Center, Memphis, TN 38103, USA; 3 Interdisciplinary Research Laboratory (LII), Nanostructures and Biomaterials Area, National School of Higher Studies (ENES), National Autonomous University of Mexico (UNAM), Leon 37684, Guanajuato, Mexico; 4 College of Dentistry, Prince Sattam Bin Abdulaziz University, Al-Kharj 11942, Saudi Arabia; 5 Department of Restorative Dental Science, College of Dentistry, King Saud University, Riyadh 11545, Saudi Arabia

**Keywords:** acid etching, hydrofluoric acid, lithium disilicate, composite resin

## Abstract

Background: This in vitro study compared the effects of four commercial hydrofluoric acid etchants with different nominal concentrations on the surface roughness of a 3D-printed lithium disilicate-filled polymer resin, evaluated after post-polymerization without debinding or sintering. Methods: Seventy-five specimens were randomly assigned to five groups (*n* = 15): G1, 4.5% hydrofluoric acid (IPS Ceramic Etching Gel, Ivoclar); G2, 4% hydrofluoric acid (Porcelain Etchant, Bisco); G3, 9.5% hydrofluoric acid (Porcelain Etchant, Bisco); G4, 9% hydrofluoric acid (Porcelain Etch, Ultradent); and G5, an unetched control. Etchants were applied for 20 s. Areal surface roughness (Sa) was measured using non-contact optical profilometry. Data were analyzed using Levene’s test, conventional and Welch’s analyses of variance, and Games–Howell pairwise comparisons (α = 0.05). Results: Both analyses of variance identified significant differences among groups (*p* < 0.001). All pairwise comparisons were statistically significant (*p* < 0.001). Mean Sa values were 0.1558 ± 0.0159 µm for G5, 0.3159 ± 0.0313 µm for G1, 0.5093 ± 0.0467 µm for G2, 0.7243 ± 0.0409 µm for G3, and 0.9365 ± 0.0408 µm for G4. The 9% Ultradent etchant produced the greatest roughness, approximately six times the control value. Conclusions: All etchants significantly increased surface roughness. However, the ranking did not follow nominal acid concentration, and differences in commercial formulations prevent attribution of the observed effects to concentration alone. These findings characterize surface modification of a 3D-printed lithium disilicate-filled polymer resin and should not be generalized to conventional lithium disilicate ceramics. Further studies are needed to determine whether these topographic changes improve subsequent adhesive bond strength or long-term durability.

## 1. Introduction

Surface conditioning of indirect restorative materials is intended to convert a relatively inert, smooth substrate into a receptive one for adhesive luting [1,2]. In clinical dentistry, hydrofluoric acid etching is used chiefly to condition the intaglio (fitting) surface of glass-matrix and resin-matrix ceramic restorations, such as veneers, inlays, onlays and crowns, before adhesive cementation, and to prepare fractured ceramic surfaces for intraoral repair; followed by silanization, it is the most widely accepted conditioning protocol for silica-based ceramics [3,4]. For glass-matrix ceramics, hydrofluoric acid selectively dissolves the glassy phase and exposes the crystalline framework, producing a network of micropores and channels into which luting resin can flow; a silane coupling agent then provides chemical continuity between the silica of the conditioned surface and the resin matrix [4,5]. The topographic consequence of this reaction is an increase in surface roughness, which enlarges the wettable area available for resin infiltration and creates the undercuts responsible for micromechanical interlocking.

This benefit is not monotonic. Etching that proceeds beyond the point at which resin can infiltrate leaves a deep, friable porous layer that is incompletely penetrated by the luting agent and that removes the silica-rich phase on which silanization depends. Increasing etching duration on lithium disilicate raises roughness while progressively reducing flexural strength [6], and roughness and wettability rise together with etching time in a manner that eventually widens pores into irregular grooves [5]. Roughness and bond strength also respond to different components of the protocol: in lithium disilicate conditioned with hydrofluoric acid, roughness was influenced by both acid concentration and exposure time, whereas bond strength was influenced only by exposure time [7]. Comparable dissociations have been reported for other glass-matrix and hybrid substrates, in which the conditioning protocol producing the greatest topographic change was not the one producing the highest or most durable bond [8,9]. Surface roughness is therefore best understood as a descriptor of the conditioned surface rather than as a measure of adhesion.

Digital dentistry incorporates additive and subtractive manufacturing approaches for processing different classes of restorative materials [10,11,12,13,14,15]. Developments in dental polymers include photopolymerizable resins containing inorganic fillers within an organic matrix [16,17,18,19]. The material evaluated in the present study is a 3D-printed lithium disilicate polymer resin, and its commercial designation should not be interpreted as indicating a fully ceramic composition. Specimens underwent light post-polymerization without debinding or sintering; therefore, the organic polymer matrix remained part of the tested substrate. This processing route differs from the additive manufacturing of lithium disilicate glass-ceramics, in which subsequent thermal treatment removes the organic binder and consolidates the ceramic material [20,21,22,23,24]. Accordingly, the specimens evaluated here should be described as a 3D-printed lithium disilicate-filled polymer resin in the post-cured, non-sintered state. Although previous investigations have examined bonding to printed, milled, and pressed lithium disilicate ceramics [22], their findings cannot automatically be extrapolated to this resin-based substrate. Its response to hydrofluoric acid must be interpreted in relation to the combined presence of an organic matrix and inorganic filler particles. This distinction provides the rationale for directly evaluating the surface changes produced by different commercial etchants on the tested material.

Commercial hydrofluoric acid etching gels are formulated and timed for pressed or milled glass-ceramics. Whether they produce comparable topographic change on a 3D-printed lithium disilicate-filled polymer resin, and whether the differences between commercial products track their stated acid concentrations, has not been characterized. Previous investigations of hydrofluoric acid etching have concerned pressed, milled or sintered lithium disilicate glass-ceramics [5,6,7,22]; to the authors’ knowledge, no study has compared commercial etching gels on a lithium disilicate-filled printed resin in the post-cured, non-sintered state, in which the acid-soluble phase is a dispersed filler within a polymer matrix rather than a continuous glassy matrix. The response of this filled printed resin to commercial etching gels has not been characterized, and that characterization is a prerequisite for selecting conditioning protocols rationally in subsequent bond strength and durability studies. The purpose of this in vitro study was to compare the areal surface roughness produced by four commercially available hydrofluoric acid etching gels of differing nominal concentration, each applied for the manufacturer-recommended 20 s, on a 3D-printed lithium disilicate-filled polymer resin tested in the post-cured, non-sintered state. The null hypothesis was that surface roughness would not differ among the four etching gels and an unetched control.

## 2. Materials and Methods

### 2.1. Specimen Fabrication

Disk-shaped specimens 10 mm in diameter and 2 mm thick were designed in computer-aided design software (Tinkercad; Autodesk Inc., San Francisco, CA, USA) and exported in standard tessellation language format. Files were prepared in slicing software (Chitubox; CBD-Tech, Shenzhen, China) with a layer thickness of 50 µm and a build orientation of 0° relative to the build platform, with supports placed on the base surface. The exposure time was 2.1 s per layer, with 6 initial layers exposed for 30 s; the printer’s 405 nm light source had an irradiance of 2.22 mW/cm^2^, with a lift distance of 6 mm and lift speed of 60 mm/min. Specimens were printed by a single operator on a liquid crystal display vat photopolymerization printer (Sonic Mini; Phrozen Technology, Hsinchu, Taiwan) from a lithium disilicate-filled ultraviolet-polymerizable resin (Lithium Disilicate UV Resin, 385–405 nm; Tethon 3D, Omaha, NE, USA). The resin was agitated manually for 5 min before printing. The manufacturer supplies this material for research and development use; it is not cleared as a clinical restorative material. According to the manufacturer, the material is an investigational, research-grade photopolymer loaded with lithium disilicate and intended for ceramic additive manufacturing with subsequent sintering; its filler content, particle size distribution and monomer composition are not disclosed [25].

After printing, specimens were air-dried to remove excess resin and cleaned with 91% isopropyl alcohol wipes until no residual uncured resin remained. Post-polymerization was performed for 20 min in a curing unit (Cure V2; Phrozen Technology, Hsinchu, Taiwan) equipped with ultraviolet light-emitting diodes at 365, 385, and 405 nm and a rotating platform. The specimens were evaluated as a 3D-printed lithium disilicate-filled polymer resin after light post-polymerization, without debinding or sintering. The test surface received no grinding or polishing; all measurements were made on the as-printed, post-cured surface. Seventy-five specimens were produced and randomly allocated to five groups of 15 by drawing lots from an opaque container (Table 1).

### 2.2. Surface Conditioning

Four commercial hydrofluoric acid etching gels were evaluated (Table 2): 4.5% hydrofluoric acid (IPS Ceramic Etching Gel; Ivoclar Vivadent, Schaan, Liechtenstein), 4% hydrofluoric acid (Porcelain Etchant 4%; Bisco, Schaumburg, IL, USA), 9.5% hydrofluoric acid (Porcelain Etchant; Bisco, Schaumburg, IL, USA), and 9% hydrofluoric acid (Porcelain Etch; Ultradent Products, South Jordan, UT, USA). Specimens in the control group received no surface conditioning.

According to the safety data sheets, hydrofluoric acid is the only hazardous ingredient declared for each gel: 4 to <7% for IPS Ceramic Etching Gel [26]; an aqueous hydrofluoric acid solution at 5–10% of the mixture for Porcelain Etchant 4% [27]; an aqueous hydrofluoric acid solution at 10–30% of the mixture, with <1% sodium fluoride, for Porcelain Etchant 9.5% [28]; and <10% for Porcelain Etch [29]. Thickeners, buffering agents, pH and viscosity are not disclosed beyond the descriptors in Table 2, so the formulation differences between products cannot be specified further, and differences in roughness cannot be attributed to hydrofluoric acid concentration alone.

Each gel was applied to the test surface in a uniform layer for 20 s, the interval specified by the manufacturers for lithium disilicate. This interval was chosen because no etching protocol is specified for this filled resin and clinicians would be expected to apply the lithium disilicate protocol; holding time constant isolates the effect of the commercial gel, but it does not identify the optimal etching time for this material, which may differ because the acid-soluble phase is a dispersed filler rather than a continuous glassy matrix. Conditioning was performed by a single operator. All specimens were then rinsed with an air–water spray for 60 s, cleaned in an ultrasonic bath for 10 min to remove residual reaction precipitate, and placed in a desiccator for 1 h. Specimens were subsequently stored dry in individual containers for 24 h before measurement.

### 2.3. Surface Roughness Measurement

**Figure 1:**
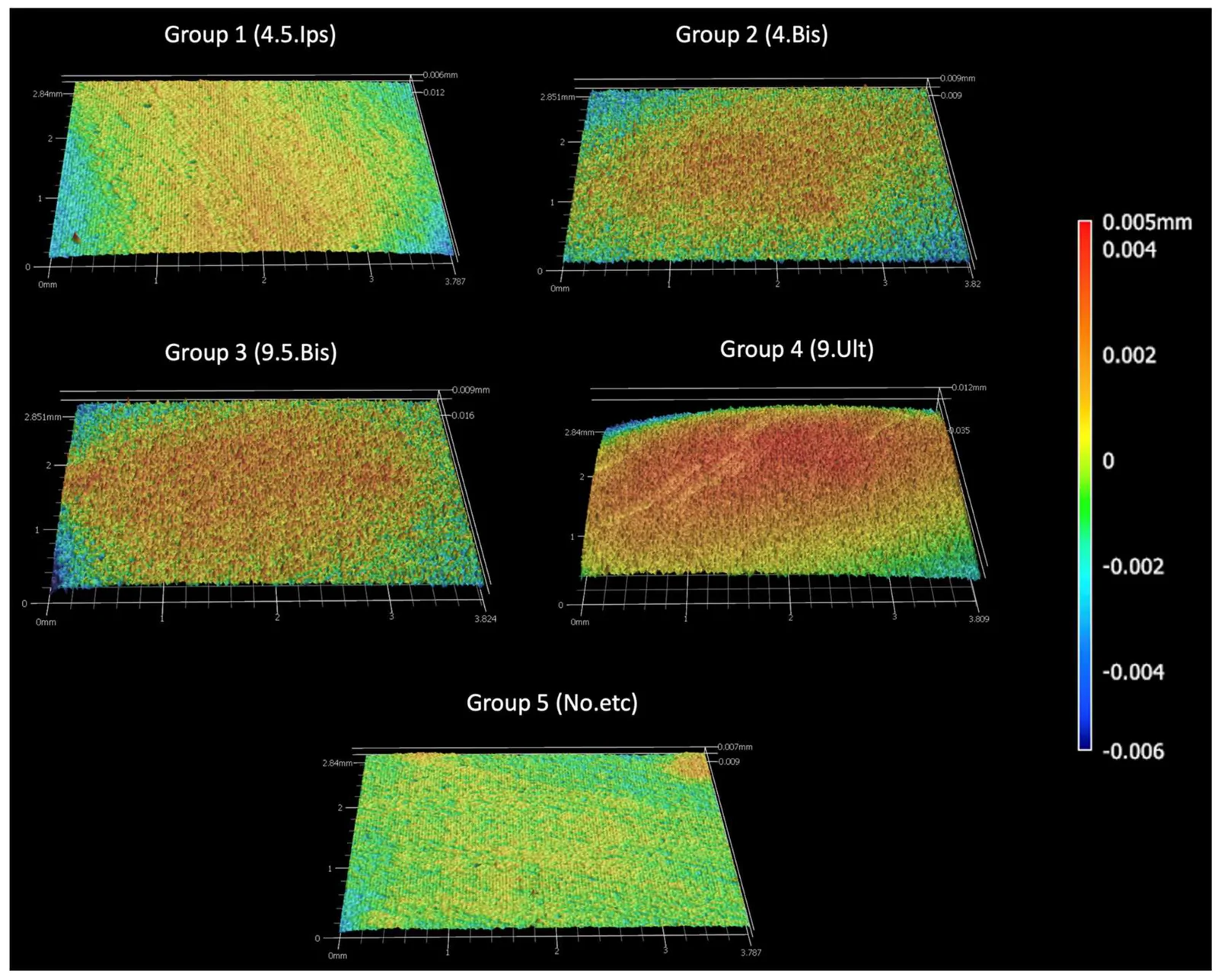
Representative three-dimensional surface topography reconstructions of the 3D-printed lithium disilicate-filled polymer resin after each conditioning protocol, acquired by non-contact optical profilometry (field of view: 3.79 × 2.84 mm). (**Group 1**) G1, 4.5% hydrofluoric acid (IPS Ceramic Etching Gel, Ivoclar); (**Group 2**) G2, 4% hydrofluoric acid (Porcelain Etchant 4%, Bisco); (**Group 3**) G3, 9.5% hydrofluoric acid (Porcelain Etchant, Bisco); (**Group 4**) G4, 9% hydrofluoric acid (Porcelain Etch, Ultradent); (**Group 5**) G5, unetched control. Lateral axes are in mm. The color scale represents surface height relative to the mean reference plane, in μm (from −6 to +5 μm). Note that vertical visualization range of topography reconstructions is not equivalent to Sa magnitude, due to image scaling.

Surface topography was recorded with a non-contact three-dimensional optical profilometer (ZeGage Pro; Zygo Corp., Middlefield, CT, USA) using a 5.5× objective with 0.75× zoom and a 1600 × 1200 measurement array, giving a field of view of 3.79 × 2.84 mm and a lateral sampling interval of 2.37 µm. Ten fields of 3.79 × 2.84 mm (about 10.8 mm^2^ each), which cannot be non-overlapping on a 10 mm disc (about 78.5 mm^2^), were recorded on the test surface of each specimen and the values were averaged. Each height map was levelled, form was removed by form-removal before parameter computation, and a Gaussian S-filter was applied in accordance with ISO 25178-3 [30]. Areal roughness Sa (ISO 25178-2 [31]) was selected instead of the profile parameter Ra because it is computed over the entire measured field rather than along a single trace, and therefore represents the spatially heterogeneous, non-directional relief produced by acid etching while being less sensitive to the position and orientation of an individual profile relative to the printing layer lines. Non-contact optical profilometry was preferred over atomic force microscopy (AFM) because it records a field of several square millimeters in a single acquisition (3.79 × 2.84 mm here), so that Sa represents the etched surface as a whole rather than a local region, and because the height variation of the etched as-printed surfaces, of the order of several micrometers, approaches the vertical range of typical AFM scanners, whose lateral scan areas are generally limited to about 100 × 100 µm [32]. The S-filter removes lateral components shorter than its nesting index, including instrument noise, before Sa is computed. The same filter setting was applied to all groups. Sa was computed for each full measurement field after levelling. The three-dimensional reconstructions in Figure 1 display the height maps as acquired, before form removal and filtering, and therefore include residual specimen form and a larger height range than the surfaces from which Sa was computed.

### 2.4. Statistical Analysis

Sample size was determined a priori in G*Power 3.1 (Heinrich Heine University, Düsseldorf, Germany) for a one-way analysis of variance with five groups, an effect size f of 0.40, corresponding to a large effect according to Cohen’s conventions and chosen based on pilot data, and α of 0.05. A total sample of 75 specimens yielded a power of approximately 0.77. This value is slightly below the conventional target of 0.80, which represents a minor limitation of this study; nevertheless, the between-group differences observed were considerably larger than the effect size assumed in the calculation, so the risk of a type II error for the reported comparisons is low. Analyses were performed in statistical software (SPSS Statistics, v30.0; IBM Corp., Armonk, NY, USA). Distributional assumptions were assessed with the Shapiro–Wilk test within each group and homogeneity of variance with the Levene test. Because variances were unequal, the Welch analysis of variance was used as the primary omnibus test and pairwise comparisons were made with the Games–Howell procedure, which does not assume equal variances. The proportion of variance explained was expressed as η^2^ with a 95% confidence interval. The significance level was set at α = 0.05. The conventional one-way analysis of variance is reported for comparison only; because variances were unequal, inference is based on the Welch test and the Games–Howell comparisons, and the agreement of the two omnibus tests indicates that the conclusions are not sensitive to the equal-variance assumption. The ten field values of each specimen were averaged before analysis, so that the specimen was the statistical unit (*n* = 15 per group) and the fields were not treated as independent observations. The effect size f = 0.40 corresponds to the conventional threshold for a large effect [33] and was computed with G*Power [34].

## 3. Results

Descriptive statistics are presented in Table 3. Values within each group were consistent with a normal distribution (Shapiro–Wilk *p* ≥ 0.142), and the Levene test indicated unequal variances across groups (*p* = 0.017). The Welch analysis of variance showed a significant difference among groups (F = 1587.12, *p* < 0.001), with η^2^ of 0.98 (95% CI 0.98 to 0.99). The conventional one-way analysis of variance agreed (F = 1080.91, *p* < 0.001).

Mean Sa was 0.16 ± 0.02 µm for the unetched control, 0.32 ± 0.03 µm for the 4.5% gel, 0.51 ± 0.05 µm for the 4% gel, 0.72 ± 0.04 µm for the 9.5% gel, and 0.94 ± 0.04 µm for the 9% gel. Games–Howell comparisons showed that all 10 pairwise differences were significant (*p* < 0.001). Every conditioned group exceeded the unetched control, and the group order by mean Sa was 9% > 9.5% > 4% > 4.5% > unetched. The 9% gel produced a mean Sa 6.01 times that of the unetched control, and the 4.5% gel produced a mean Sa 2.03 times that of the unetched control. The coefficient of variation within groups ranged from 4.4% in the 9% group to 10.2% in the unetched control.

The ranking did not follow nominal acid concentration. The 9% gel produced a mean Sa 0.21 µm higher than the 9.5% gel, a difference of 29% (*p* < 0.001). Within the two gels supplied by a single manufacturer, Sa rose with concentration, from 0.51 ± 0.05 µm at 4% to 0.72 ± 0.04 µm at 9.5% (*p* < 0.001).

Representative topographic reconstructions are shown in Figure 1. The unetched surface (Figure 1, Group 5) showed low-amplitude height variation across the measured field, consistent with the layer banding of the as-printed surface. Conditioned specimens showed greater height amplitude and a more irregular relief as Sa increased. The vertical range of the reconstructions, approximately ±6 µm, is substantially greater than the Sa values, so residual specimen form contributes visibly to the appearance of the reconstructions, most evidently in Figure 1 Group 4. The reconstructions are presented as acquired; the filtering applied before computation of Sa is described in the Materials and Methods.

## 4. Discussion

This study demonstrated that all hydrofluoric acid etching protocols significantly increased the surface roughness of the tested material compared to the unetched control group. This finding is consistent with previous literature establishing that untreated ceramic surfaces are generally smooth and homogeneous, whereas acid etching creates a distinctly porous and irregular topography [1,4]. In glass-ceramics, this topography results from selective dissolution of the glassy matrix, which exposes the lithium disilicate crystals [5,35]. That mechanism cannot be transferred directly to the present material, in which the continuous phase is a cross-linked organic polymer that is not expected to be appreciably dissolved by hydrofluoric acid during a 20 s exposure, whereas the dispersed lithium disilicate filler is the acid-susceptible, silica-based phase. The increase in Sa is therefore more plausibly explained by partial dissolution of filler particles exposed at the surface, by loss of particles whose interface with the polymer has been attacked, leaving pits in the matrix, and possibly by retained reaction products. Because neither SEM nor EDS was performed, this mechanism remains a hypothesis to be tested. The two higher-concentration gels (9% and 9.5%) produced greater roughness than the two lower-concentration gels (4% and 4.5%), in general agreement with a previous report on lithium disilicate glass-ceramic, in which 5% hydrofluoric acid left a smoother, less conditioned surface than 10% and the commercial brand of the etchant also influenced bond strength [36]; however, the ranking did not strictly follow nominal concentration, because the 9% gel exceeded the 9.5% gel, and the differences therefore cannot be attributed to concentration alone. In glass-ceramics, etching increases the number of irregularities, channels and pores, which elevates roughness measurements [6]; whether comparable features form in this filled resin was not examined directly.

Additive manufacturing techniques, such as digital light processing and stereolithography, have emerged as highly efficient alternatives to traditional subtractive milling, offering the ability to produce highly accurate, patient-specific dental prostheses with complex geometries. These layer-by-layer manufacturing processes significantly minimize material waste and production costs while maintaining high standards of marginal fit and anatomical precision [37]. As these digital workflows continue to mature, the dental industry has pivoted toward developing specialized 3D-printable materials engineered for long-term applications, heavily relying on the continuous optimization of the printing parameters and the formulation of novel photopolymerizable resins [38,39].

Three-dimensional printed resins represent an important category of materials in digital dentistry, in which a photopolymerizable organic matrix enables layer-by-layer fabrication. Their properties depend on resin formulation and processing conditions, including layer thickness and post-polymerization treatment. Within this context, the experimental material evaluated in the present study should be identified as a 3D-printed lithium disilicate-filled polymer resin. The manufacturer describes this formulation as a ceramic-filled photopolymer, indicating that lithium disilicate is incorporated into a resin-based system. As specified in the present methodology, the specimens underwent light post-polymerization without debinding or sintering; therefore, the polymeric matrix remained a constituent of the tested material. The presence of lithium disilicate particles does not make these specimens equivalent to fully processed lithium disilicate glass-ceramics. Consequently, the observed surface changes characterize the response of a 3D-printed lithium disilicate-filled polymer resin, and their interpretation must account for both the organic matrix and the inorganic filler. These findings should not be directly extrapolated to conventional pressed, milled, or sintered lithium disilicate ceramics.

Initially, the focus of additive manufacturing in dentistry centered on polymer-based composites reinforced with various fillers to mimic natural dentition, but the search for superior aesthetics and mechanical longevity has driven the emergence of hybrid materials [36,38]. Understanding the mechanical properties of these novel 3D-printed restorative materials is critical for their predictable application under functional occlusal loads. The structural integrity, including flexural strength and fracture resistance, is significantly influenced by specific manufacturing parameters such as printing orientation, layer thickness, and post-curing conditions [39,40,41].

A prominent example of this innovation is the development of 3D-printed lithium disilicate and silicate glass-ceramics, which strive to combine the facile processability of light-cured resins with the exceptional optical and mechanical properties of traditional ceramics [39,42]. The literature shows that excessive surface roughness or over-etching has a detrimental weakening effect on the restorative material [6]. In such sintered glass-ceramics, more concentrated acid would be expected to dissolve the glassy matrix more aggressively and expose more of the crystalline network; this mechanism does not apply directly to the non-sintered resin tested here. The layer-wise deposition and subsequent thermal processing, such as debinding and sintering, result in a unique microstructure that fundamentally differs from conventionally pressed or milled ceramics. While these novel printable glass-ceramics present new possibilities for highly aesthetic restorations, their distinct structural density introduces new challenges in standardizing their clinical handling. Consequently, more studies are required to fully understand how these microstructural differences impact the long-term behavior of the final restorations. External mechanical surface modifications, such as those performed during laboratory finishing or adjustments, have also been shown to directly impact the surface roughness and the maximum fracture force that these printed components can withstand [43].

Traditional surface conditioning protocols prioritize the creation of a high-energy, retentive substrate through mechanical and chemical treatments, such as alumina air abrasion, hydrofluoric acid etching, and the application of silane coupling agents [41,44]. Although increased surface roughness is beneficial for enhancing wettability and providing a significantly larger surface area for resin infiltration, the creation of excessively deep and wide porosities might compromise the structural integrity and flexural strength of the substrate [43].

From an application standpoint, the roughness produced by etching matters in two opposite ways. On the intaglio surface, a higher Sa enlarges the area available for wetting and infiltration by the luting agent and provides the micro-retentive irregularities required for micromechanical interlocking, which, together with silanization, underlies durable resin bonding [3,4]; roughness is nevertheless a descriptor of the conditioned surface and not a substitute for bond strength testing [7,8,9]. Conversely, any etched surface left exposed to the oral environment, for example at restoration margins or after intraoral repair, is relevant biologically: a threshold Ra of approximately 0.2 µm has been proposed above which plaque retention increases [45]. In the present study, the unetched surface (Sa 0.16 µm) lay below this value, whereas all etched groups exceeded it, although Sa and Ra are not numerically interchangeable. Etching of this material should therefore be confined to the bonding surface.

Hydrofluoric acid is not the only conditioning option for resin-based printed materials. Airborne-particle abrasion with alumina, alone or followed by silane, is widely used for resin-matrix CAD/CAM and printed materials, and in several recent in vitro studies on printed permanent resins it produced greater roughness and higher bond strength than hydrofluoric acid etching [45,46,47,48,49]; one study concluded that hydrofluoric acid etching should not be recommended for all resin-based CAD/CAM materials [46]. Hydrofluoric acid is also hazardous, which has motivated the search for alternative conditioning methods [4]. The rationale for evaluating hydrofluoric acid on the present material is its lithium disilicate filler, a silica-based phase that hydrofluoric acid dissolves selectively, whereas airborne-particle abrasion acts mechanically and non-selectively on matrix and filler alike. The present study therefore does not show that hydrofluoric acid is superior to other treatments; it characterizes how commercial hydrofluoric acid gels modify this specific substrate, and direct comparisons with airborne-particle abrasion, including bond strength, are required.

The baseline roughness of the unetched control (Sa 0.16 µm) reflects the as-printed surface. Specimens were printed at 0° relative to the build platform with 50 µm layers, post-polymerized for 20 min at 365, 385 and 405 nm, and measured on the upper surface opposite the supports, without grinding or polishing. Build orientation, layer thickness and post-polymerization conditions influence the surface texture, degree of conversion and mechanical properties of printed resins [39,40,41], and the degree of conversion of the near-surface polymer may in turn affect how readily filler particles are exposed or lost during etching. Because these parameters were held constant, their contribution to the response to etching was not evaluated, and the results apply to the printing and post-curing conditions used here.

The roughest surface, produced by the 9% gel, should not be read as the most favorable one. In glass-ceramics, more aggressive etching reduces flexural strength [6], and in a filled resin, attack at the filler–matrix interface could reduce filler retention and weaken the near-surface layer. Flexural strength, filler retention, bond strength, wettability and durability after aging were not measured, so the present data do not support conclusions about restoration longevity, fracture resistance or long-term bonding. The findings are specific to this material and processing route and should not be generalized to other printed resins, whose filler type, content and matrix chemistry differ, or to fully processed lithium disilicate ceramics. Establishing whether the protocol producing the highest roughness also provides superior and durable adhesion would require microtensile or shear bond strength testing with a silane-containing luting system before and after thermocycling, combined with failure-mode analysis, SEM/EDS of the etched surfaces and flexural strength testing. To separate the independent effects of concentration, formulation and time, a factorial design using experimental gels of identical base formulation at several hydrofluoric acid concentrations, crossed with several etching times (for example, 20, 40 and 60 s) and compared with the commercial products, would be needed.

Despite the promising aesthetic and clinical potential of 3D-printed hybrid resins and lithium disilicate ceramics, there is a distinct lack of comprehensive studies evaluating the specific effects of various surface conditioning protocols on their unique topographies [39]. Because the chemical composition, filler distribution, and layer-by-layer structural density of these additively manufactured materials differ significantly from conventionally fabricated ceramics, standard etching times, acid concentrations, or blasting pressures may not yield the same surface pattern outcomes [42]. This microstructural variability can lead to inconsistent surface degradation or inadequate micromechanical porosities when traditional adhesive guidelines are applied [41]. Therefore, it is imperative to systematically evaluate and compare the efficacy of different hydrofluoric acid etchants and mechanical treatments on these specific printable substrates [44]. Establishing tailored and standardized clinical guidelines for surface conditioning will ultimately ensure the predictable integration and mechanical stability of these novel 3D-printed restorations [43].

Roughness was quantified at the micrometer scale by optical profilometry; nanoscale features within the etch pits, which AFM could resolve, were not characterized. Moreover, all Sa values were obtained with a single filtering configuration, and the height maps were not available for re-analysis; the sensitivity of the absolute Sa values and of the group ranking to alternative S-filter nesting indices therefore could not be assessed and remains unverified. Finally, only Sa was reported; amplitude and hybrid parameters such as Sq, Sz and Sdr, which would describe peak-to-valley height and the developed interfacial area available for bonding, could not be calculated retrospectively because neither the height maps nor the per-field instrument reports were retained. Sz and Sdr carry information that Sa does not, namely the extreme peak-to-valley height and the increase in true surface area, and their absence limits the description of the etched topography. This study has limitations. Surface characterization was restricted to profilometric areal roughness; scanning electron microscopy and energy-dispersive spectroscopy were not performed, so the micromorphological features underlying the Sa differences, such as dissolution of lithium disilicate filler particles, pits left by dislodged particles, or residual reaction precipitates, were not directly observed. In particular, the higher Sa produced by the 9% gel (Ultradent) than by the 9.5% gel (Bisco), despite its lower nominal concentration, cannot be explained from the present data. Because the two products differ in formulation as well as in concentration, a difference in effective etching activity, or in the amount of reaction precipitate retained on the surface after cleaning, is a plausible explanation, but it remains a hypothesis. In addition, the a priori power of the design (0.77) was slightly below the conventional target of 0.80. Future studies should combine profilometry with scanning electron microscopy and energy-dispersive spectroscopy of the etched surfaces, and with bond strength testing, to establish the mechanism of these differences and their clinical relevance.

## 5. Conclusions

Based on the findings of this in vitro study, the following conclusions can be drawn regarding the surface conditioning of the tested 3D-printed lithium disilicate-filled polymer resin:

1. All four hydrofluoric acid etching gels significantly increased the surface roughness of this novel 3D-printed lithium disilicate UV resin compared to an untreated surface.
2. The two higher-concentration gels (9% and 9.5%) produced significantly greater surface roughness than the two lower-concentration gels (4% and 4.5%); however, the ranking did not strictly follow nominal concentration, and differences between commercial formulations prevent attribution of the effect to concentration alone.
3. Among the tested commercial etchants, the 9% hydrofluoric acid (Ultradent) application for 20 s produced the highest measurable surface roughness. Whether this roughness benefits bonding, or instead weakens the polymer matrix or reduces filler retention, was not evaluated.

Bond strength, durability and fracture resistance testing are required before any of these etching protocols can be recommended for this material, or the material can be considered for definitive restorations.

## Figures and Tables

**Table 1:** Summary of groups.

Group	Conditioning	Nominal Hydrofluoric Acid Concentration (%)	Number
G1	IPS Ceramic Etching Gel	4.5	15
G2	Porcelain Etchant 4%	4	15
G3	Porcelain Etchant	9.5	15
G4	Porcelain Etch	9	15
G5	None (control)	—	15

**Table 2:** Etching gels evaluated.

Property	IPS Ceramic Etching Gel	Porcelain Etchant 4%	Porcelain Etchant	Porcelain Etch
Manufacturer	Ivoclar Vivadent, Schaan, Liechtenstein	Bisco, Schaumburg, IL	Bisco, Schaumburg, IL	Ultradent Products, South Jordan, UT
Nominal concentration (%)	4.5	4	9.5	9
Color	Red	Orange	Blue	Yellow
Consistency	Viscous gel	High-viscosity gel	High-viscosity gel	High-viscosity gel
Manufacturer stated application	Intraoral use, buffered; 20 s	Intraoral use with rubber dam; 20 s	Intraoral use with rubber dam; 20 s	Extraoral use; 20 s

**Table 3:** Descriptive statistics of areal surface roughness Sa (μm) for all experimental groups; different superscript letters indicate significant pairwise differences at α = 0.05.

Group	*n*	Mean	SD	SE	95% CI Lower	95% CI Upper
G1 (4.5% Ivoclar	15	0.3159 ^d^	0.03128	0.00808	0.2986	0.3333
G2 (4% Bisco)	15	0.5093 ^c^	0.04669	0.01205	0.4835	0.5352
G3 (9.5 Bisco)	15	0.7243 ^b^	0.04093	0.01057	0.7016	0.7469
G4 (9% Ultradent)	15	0.9365 ^a^	0.04084	0.01054	0.9139	0.9591
G5 (control)	15	0.1558 ^e^	0.01586	0.00409	0.1470	0.1646

*n*, number of specimens per group; SD, standard deviation; SE, standard error of the mean; CI, confidence interval. All values are in μm. Pairwise comparisons were made with the Games–Howell procedure. Different superscript letters indicate statistically significant differences (*p* < 0.05).

## Data Availability

The original contributions presented in the study are included in the article; further inquiries can be directed to the corresponding author.
